# Widespread mosquito net fishing in the Barotse floodplain: Evidence from qualitative interviews

**DOI:** 10.1371/journal.pone.0195808

**Published:** 2018-05-02

**Authors:** David A. Larsen, Rick Welsh, Angela Mulenga, Robert Reid

**Affiliations:** 1 Department of Public Health, Food Studies and Nutrition, Syracuse University, Syracuse, New York, United States of America; 2 Independent scholar, Lusaka, Zambia; 3 Liuwa Plain National Park, African Parks Zambia, Kalabo, Zambia; Centers for Disease Control and Prevention, UNITED STATES

## Abstract

**Background:**

The insecticide-treated mosquito net (ITN) is a crucial component of malaria control programs, and has prevented many malaria cases and deaths due to scale up. ITNs also serve effectively as fishing nets and various sources have reported use of ITNs for fishing. This article examines how widespread the practice of mosquito net fishing with ITNs is.

**Methods:**

We conducted in-depth interviews with fishery personnel and traditional leadership from the Barotse Royal Establishment in Western Province, Zambia, to better understand the presence or absence of the use of ITNs as fishing nets. We then coded the interviews for themes through content analysis. Additionally we conducted a desk review of survey data to show trends in malaria indicators, nutritional status of the population and fish consumption.

**Results:**

All those interviewed reported that ITNs are regularly used for fishing in Western Zambia and the misuse is widespread. Concurrently those interviewed reported declines in fish catches both in terms of quantity and quality leading to threatened food security in the area. In addition to unsustainable fishing practices those interviewed referenced drought and population pressure as reasons for fishery decline. Malaria indicators do not show a trend in declining malaria transmission, fish consumption has dropped dramatically and nutritional status has not improved over time.

**Conclusions:**

Despite the misuse of the ITNs for fishing all those interviewed maintained that ITN distribution should continue. Donors, control programs and scientists should realize that misuse of ITNs as fishing nets is a current problem for malaria control and potentially for food security that needs to be addressed.

## Introduction

The insecticide-treated mosquito net (ITN) has saved millions of lives since its inception and prevented countless cases of malaria [1]. Prior to the development of ITNs mosquito control for malaria vectors was unavailable in most countries due to the environmental ban of DDT and a lack of alternative insecticides. The creation of pyrethroid-class insecticides allowed for the treatment of mosquito nets with an agent lethal to mosquitos but safe for humans [2]. The species of mosquito capable of malaria transmission typically seek blood meals at night when humans are indoors [3]. The mosquito net prevents a malaria-infected mosquito seeking a blood meal to bite and infect the person sleeping under an ITN and the insecticide kills the host-seeking mosquito and subsequently lowers the spread of malaria. The resultant decline in malaria transmission and child mortality is dramatic, with an estimated 17% reduction in all-cause child mortality in malaria-endemic areas [4]. Mass free distribution of ITNs have been highly successful at quickly scaling up coverage, especially in Zambia [5], and household ITN ownership is one of the most equitable public health interventions in the world [6].

Because of the benefit that ITNs provide to users and non-users alike [7,8], ITNs are viewed as a public good similar to vaccines. As such, they are commonly given freely to individuals in malaria-endemic region which has led to highly equitable ITN coverage across social strata [9]. Distribution mechanisms include receipt of an ITN through antenatal care as well as mass distribution campaigns. Although hundreds of millions of ITNs have been distributed throughout sub-Saharan Africa over the past decade [10], not all the nets are used. Analyses of survey data found that only half of children under the age of 5 and pregnant women living in houses with ITN actually used them [11]. Numerous reasons exist for individuals not using a mosquito net while sleeping, including the discomfort of the nets themselves [12]. Little has been documented in regards to what individuals do with mosquito nets when they do not sleep underneath them.

Recently news media has suggested that ITNs are being diverted from mosquito control for fishing [13], a claim which if substantiated would further understanding why malaria persists in the area despite scaled ITN coverage. Some scientists dismiss outright these types of claims [14], while others have documented the misuse of ITNs for fishing elsewhere on the shores of Lake Victoria [15], coastal Kenya [16], and throughout the world [17]. From a conventional size-selective management paradigm of fishery management mosquito net fishing (MNF) may threaten the strength of fisheries due to the small mesh size not allowing for reproduction of larger species [18–21]. Alternative fishery management paradigms, such as balanced harvest theory [22], suggest that mosquito nets used in MNF would not be problematic at all. Further complications may arise with the use of ITNs as fishing nets–the pyrethroids used in ITNs are also extremely toxic to fish and aquatic environments [23,24]. The extent to which pyrethroids leach from fishing with ITNs remains unknown, however, and further research needs to be done on how these pyrethroids interact in natural aquatic environments.

For the subsistence farmer, fish represent an important cash crop and a significant source of protein and micronutrients [25–29]. Specifically in the Barotse flood plain, fish accounts for approximately 73% of cash income [30]. Markets for fish caught in the flood plain range from local outlets to markets in Lusaka and even the Zambian cooper belt and exports markets outside of Zambia. During the late 20^th^ century the method of seine-net fishing was introduced to the area to complement traditional techniques such as woven baskets, spears and fish traps. Seine-net fishing can result in large fish catches, however under the current fisheries management paradigms the sustainability of seine fishing depends upon the size of the mesh used in the net. Controlled mesh sizes allow smaller fish and juveniles of larger species to escape. Smaller species are protected in order to support sustainable food webs. Larger species are caught above an estimated ‘length at first maturity’ in order to allow individuals to breed prior to capture. Under a balanced fishing management paradigm, the sustainability of seine-net fishing depends upon moderating fishing activity–the amount of fishing can become problematic however the mesh size of the net is not [31]. Ensuring that either small mesh nets are not used or moderating the amount of fishing both pose further challenges fishing sector in Zambia, the management of which is already challenged by low capacity due to numerous factors including a lack of storage facilities, unclear objectives in fisheries management, limited access to finances, and weak enforcement of regulations.

To determine whether mosquito net fishing with ITNs is a problem in Western Province, Zambia we first conducted qualitative interviews with 15 members of the traditional leadership in the Barotse Royal Establishment (BRE) of Western Province and 15 individuals working in Western Province with Zambian government agencies and African Parks Zambia. Following the interviews we conducted a desk review of population-based surveys to understand historical malaria trends in this area.

## Methods

### Study setting

**Fig 1:**
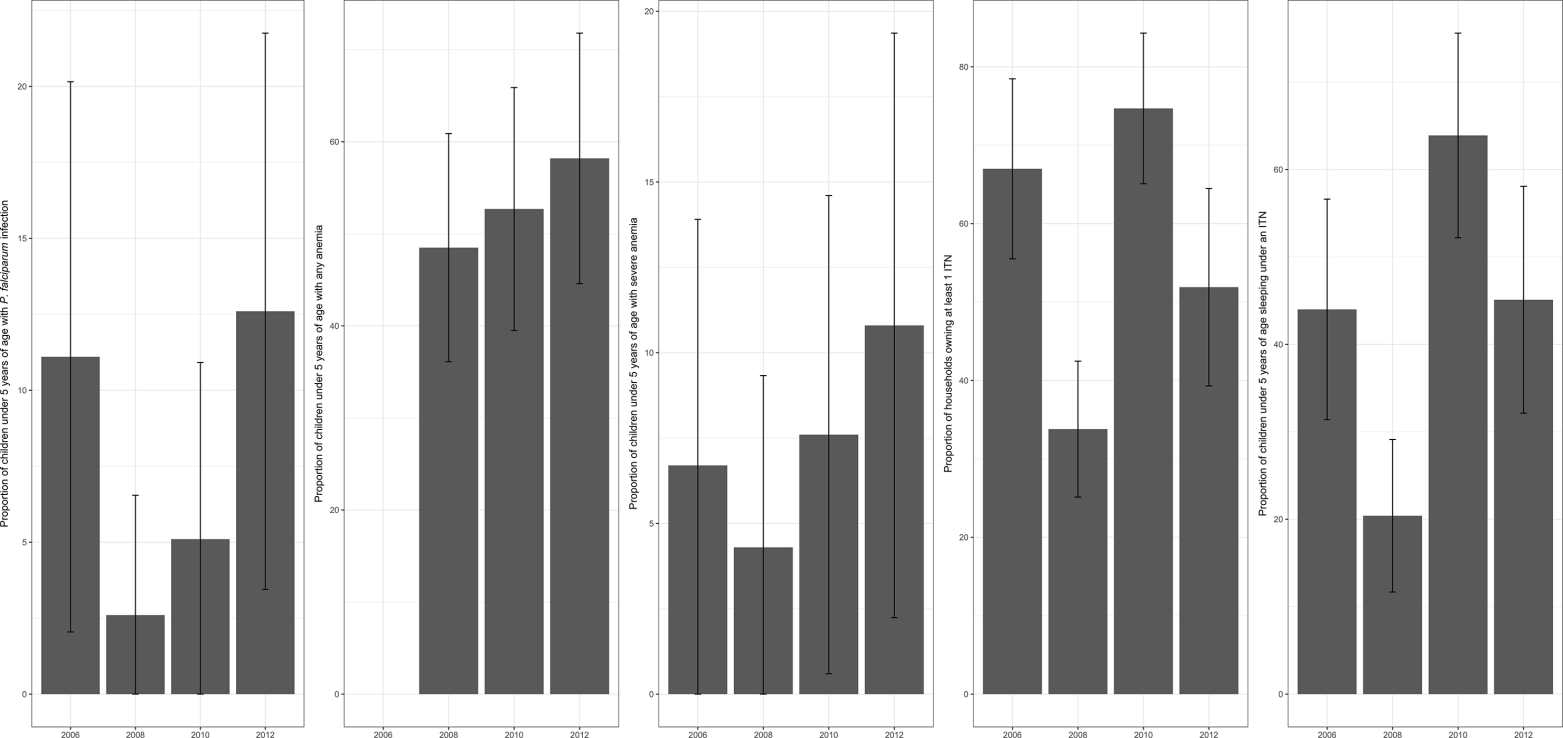
Various indicators of malaria transmission in Western Province (Barotseland), Zambia as measured by malaria indicator surveys. Malaria infection (*Plasmodium falciparum*) in children younger than 5 years of age decreased sharply in 2008 compared to 2006 but has since rebounded. Anemia levels, an indicator of nutritional status as well as malaria transmission [37], have also increased.

Western Province includes the Barotse flood plain, an area upstream from Victoria Falls where the Zambezi River and its tributaries burst banks each year and create a vast wetland perfect for breeding mosquitos. Malaria is a chief health concern, and recent efforts to control or decrease transmission have not been successful as evidenced by little change in malaria parasite prevalence in children under 5 years of age as well as anemia levels (Fig 1). The distribution of ITNs has continued throughout this time period, however, as part of the National Malaria Control Centre’s strategy to ensure an ITN for every sleeping space.

### In-depth interviews

We identified two key informants to assist us in purposefully sampling two groups charged with overseeing and managing fisheries in Western Province, Zambia: (1) the traditional leaders in the Barotse Royal Establishment (BRE); and (2) agency personnel from African Parks Zambia and the Zambian Ministry of Livestock and the Department of Fisheries. One key informant was a member of the BRE and had a personal connection with an employee of a health non-governmental organization operating in Zambia with which one of our co-authors had worked. The other key informant is a co-author of this paper (Reid) and an employee of African Parks Zambia. He has worked on this issue for a number of years and reached out to the other co-authors so a systematic investigation of the ad hoc observation of the use of ITNs as fishing nets could be advanced. African Parks Network is an international non-governmental organization that manages national parks throughout Africa.

These two groups are particularly well placed to provide critical information on this topic. The BRE is the traditional government structure of the region. The leaders range in rank from village headmen (lowest) to senior chiefs and chieftainesses (highest below The Litunga or king). Rank depends primarily on the number of villages and land over which the traditional leaders maintain control [32]. Traditional leaders in this part of Zambia have significant authority over land ownership and land use issues, and are involved actively in the management of natural resources in this region including policing the fisheries [32,33].

In addition, officials stationed in Western Province with African Parks Zambia, the Zambian Ministry of Agriculture and Livestock and the Department of Fisheries are also charged with overseeing natural resource management in the region, but from federal government and transnational park management agreement authority. Fifteen traditional leaders and 15 agency personnel were interviewed during July of 2016. The interviews were conducted using an interview guide approach with predetermined questions but following a flexible format, which allows emergent issues to be pursued. Interviews lasted approximately 30 minutes. Subjects were queried on the importance of fishing to local diets and the local economy. In addition, subjects were asked if ITNs were used as fishing nets, if so were only older nets used and what impact were the use of ITNs having, if any, on the health of local fisheries.

The interviews were recorded and transcribed. In this type of research methodology, the intent is to identify and interview those persons who can serve as information-rich cases through purposeful, non-probability, sampling. That is, the researchers seek individuals who have the specific knowledge of the issues of interest to the researcher [34].

Transcriptions were analyzed using standard qualitative analysis methods of data reduction, data display and conclusions drawing [35]. Data were “reduced” through reviewing subjects’ response to the questions in the interview guide and searching for patterns. In the case of the traditional leaders and agency personnel responses emerged regarding:

- knowledge of target fish
- technologies and techniques used for fishing including ITNs
- fishery health
- policy or management options

Once these categories had emerged from the transcript data, we displayed the data through the process of cutting and pasting quotes from the subjects within each category. This display enabled us to efficiently perceive, understand and summarize the observations, experiences and attitudes of the interviewed subject in regards to the research topic.

### Ethics approval and consent to participate

The Barotse Royal Establishment granted permission to conduct the research with traditional leaders. Individual informed consent was obtained prior to all interviews. The research protocol was approved by the Syracuse Institutional Review Board, #16–106.

### Desk review of two-stage cluster surveys

To examine temporal trends in fish consumption and child growth data were retrieved from publicly available nationally representative Demographic and Health Surveys (DHS) and Multiple Indicator Cluster Surveys (MICS) conducted in Zambia since the year 2000. The DHS ask questions about food consumption within the last 24 hours for the youngest child under the age of 5 of each mother interviewed. Other researchers have used them to examine the diversity of diet in children [36]. Protein intake among children from the 2007 DHS and 2013 DHS was disaggregated by food type and also combined to show any protein intake. Both the DHS and MICS assess child anthropometry. Stunted growth among children under the age of five was defined as two standard deviations below the World Health Organization reference population in accordance with international standards. Stunting prevalence data was available from the 2000 MICS, 2002 DHS, 2007 DHS, and 2013 DHS.

To examine temporal trends in malaria indicators we conducted a desk review of the 2006 MIS, 2008 MIS, 2010 MIS, and 2012 MIS. These datasets are not publicly available, and so figures from the published reports were used. The MIS in Zambia is powered to estimate parasite prevalence within the 9 provinces. Malaria parasite prevalence of children under the age of five was measured by microscopic diagnosis and reported in 2006, 2008, 2010 and 2012 MIS. Anemia can result from both malaria infection and malnutrition, and is an indicator of malaria interventions [37]. The prevalence of anemia in children under the age of 5 as measured by Hemocue rapid diagnostic sticks was reported in the 2008 MIS, 2010 MIS, and 2012 MIS. Any anemia was categorized as hemoglobin levels < 12.0 g / dL of blood, and moderate or severe anemia was categorized as hemoglobin levels < 9.0 g / dL of blood. Unfortunately the report for the 2015 MIS undertaken in Zambia was not available to the public at the time of writing this article (October 2017). We estimated standard errors for each measure retrieved from the report with the following equation:

$$Deft=\frac{se_{cluster}}{se_{simple}}$$

where Deft is the design effect presumed to be 2, se_cluster_ is the standard error to be estimated and se_simple_ is retrieved from the report with the typical equation for a standard error.

$$se=\sqrt{\frac{p(1-p)}{n}}$$

## Results

### In-depth interviews

**Fig 2:**
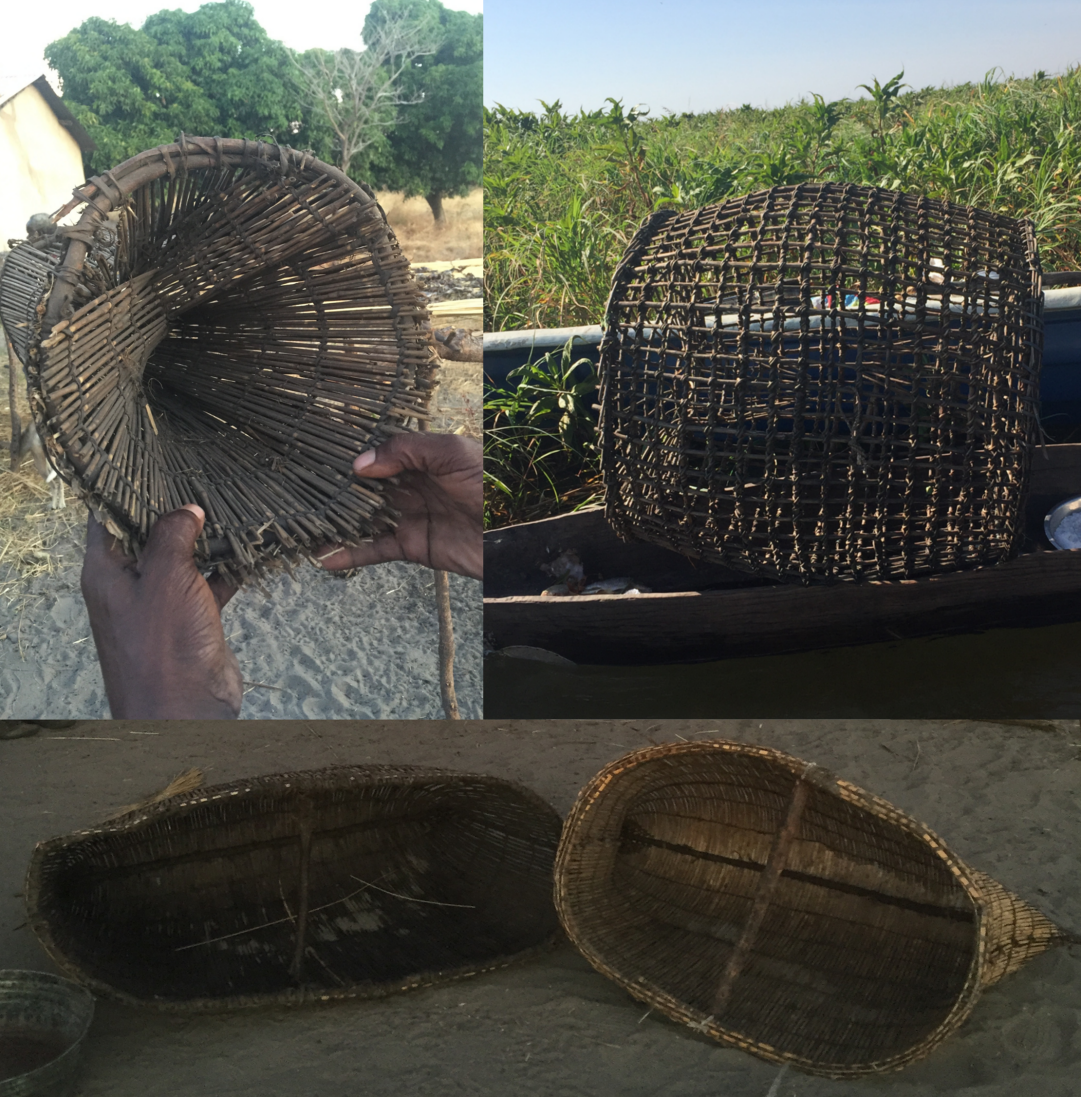
Traditional methods of fishing in the Barotse floodplain. Methods include hand-sized baskets to catch small fish (A,D), spears (B), and baskets to catch larger fish (C).

According to the traditional leaders, fish is a critically important source of protein for the people of Western Province. Traditional methods of fishing are designed to be selective to both not target entire pans as the sefa-sefa drag nets do and also allow the escape of very small juvenile fish to ensure a plentiful fish stock (Fig 2) to be sustainably utilized throughout the year. As the season changes so the methodology and utilization changes to allow this harvest to continue. Leaders went to great pains to emphasize that such methods have been in use for at least hundreds of years and had not resulted in problems with declining fish populations from overfishing. Indeed, the debate over selective- versus balanced-fishing should take into account that the indigenous fishing communities, at least in this region, seem to have employed a selective-fishing approach combined with limiting over-fishing.

**Fig 3:**
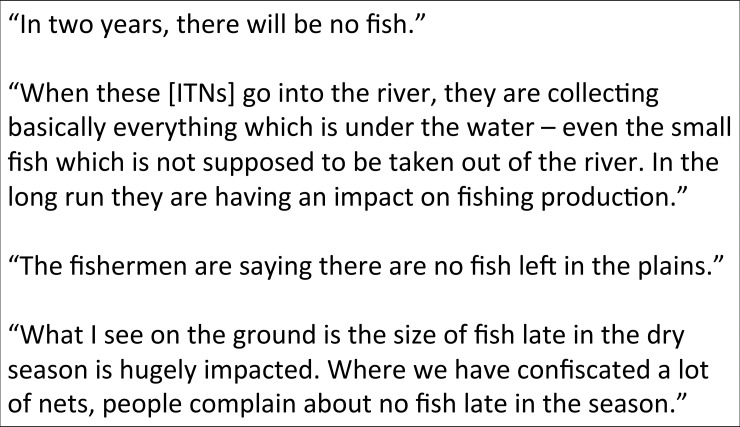
Selected quotes from interviews with traditional leaders from the BRE and agency personnel.

When asked about the current and expected future health of the fisheries, leaders were pessimistic (Fig 3). Fish populations were believed to be declining and fish prices increasing for all types. Primarily the leaders observed the decline in the size of the fish caught as well as the fewer numbers. Personnel from African Parks Zambia and the Zambian Ministry of Agriculture and Livestock and Department of Fisheries agreed that the misuse of ITNs as fishing nets were having a negative impact on fisheries in the area.

In general, the leaders attributed declining fish catches to three main factors: drought, population increase and use of very small mesh nets for fishing, including but not limited to ITNs. In fact, leaders described the use of various unorthodox materials as fishing nets in addition to ITNs such as shade cloth and even bed sheets. Also, leaders explained that the use of small mesh fishing nets preceded the distribution of ITNs. And some leaders indicated that it was primarily new arrivals to the area, rather than the long-term resident fishing populations, which introduced the small mesh fishing nets- known locally as “sefa-sefa” (to sweep). However, as ITNs became available, they were also used as sefa-sefa or incorporated into sefa-sefa, primarily as the cod-end (or bag) at the end of the drag net.

**Fig 4:**
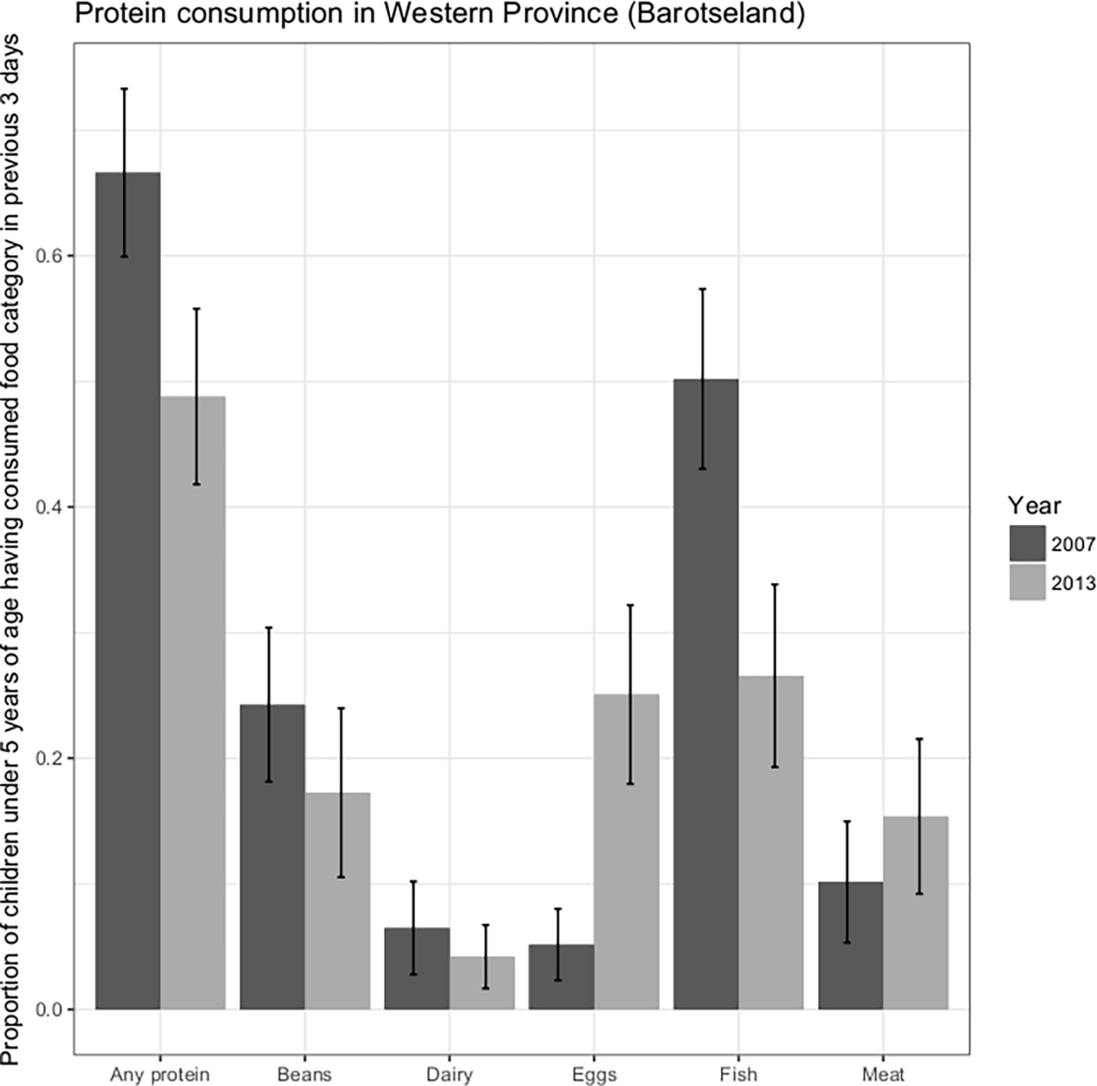
Protein and fish consumption in Western Province, Zambia. Protein consumption, and fish consumption in particular in Western Province, Zambia decreased in 2013 compared to 2007 as measured by Demographic and Health Surveys. Chi-square statistic for change in any protein = 12.7576, p = 0.0007; chi-square statistic for change in fish consumption = 19.1685, p < 0.0001.

**Fig 5:**
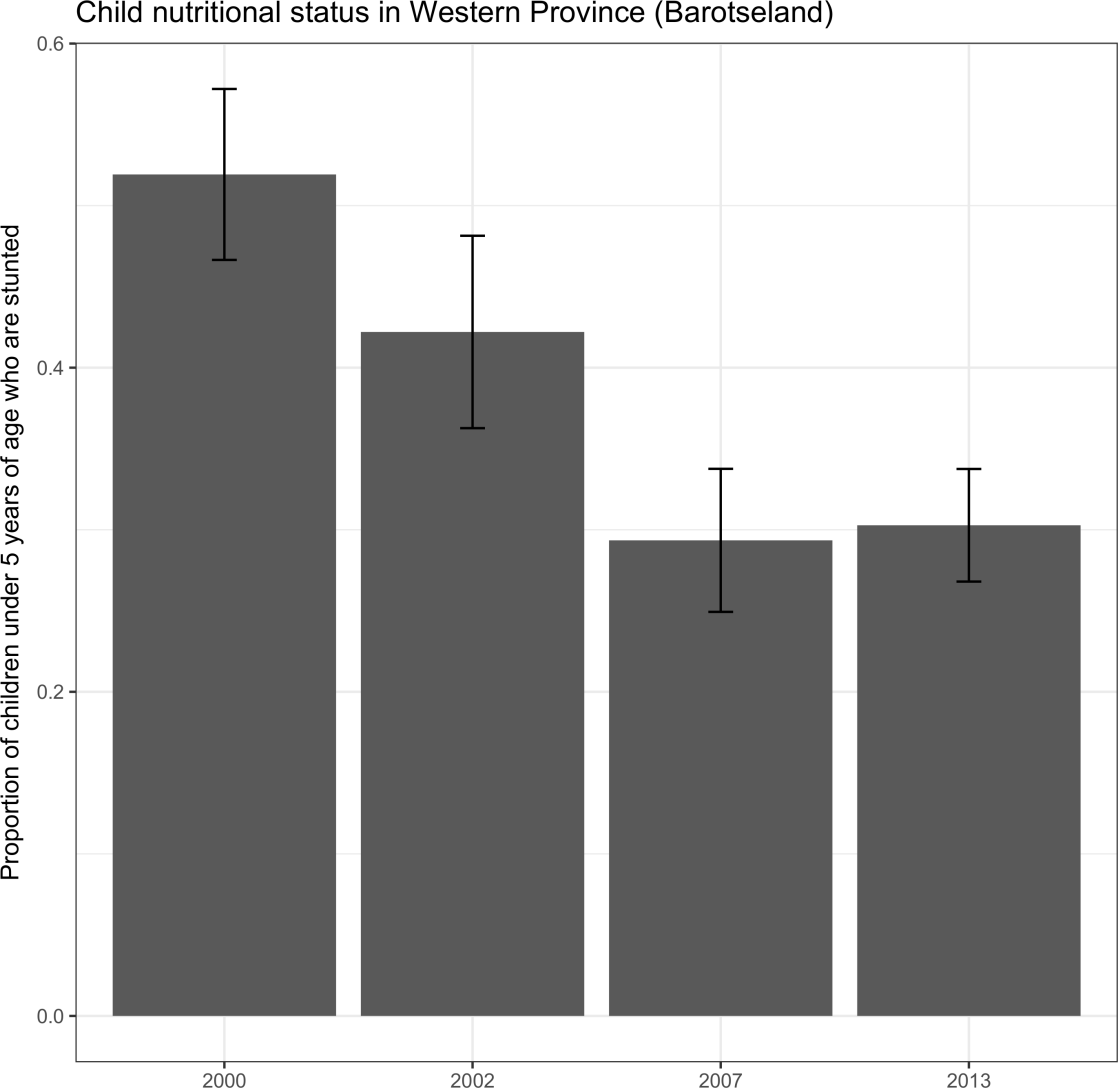
Child malnutrition in Western Province, Zambia. Child malnutrition as measured by stunted growth in Western Province, Zambia.

When asked specifically about the use of ITNs, traditional leaders and agency personnel indicated that both new nets and older nets were used, either individually, or sewn into larger nets (Figs 4 and 5). The larger nets could be composed solely of ITNs or include ITNs along with small mesh fishing nets and other materials. Leaders also observed that at times newly distributed ITNs were diverted from malaria control and used only for fishing. Other times a household might have extra ITNs such that household members could be protected from mosquitoes and the ITNs could be used for fishing. Since ITNs are distributed for free and in very large numbers, they are readily available for many uses.

Despite the number of problems raised with the use of ITNs, both traditional leaders and agency personnel were opposed to stopping their distribution. Perceived dramatic reductions in malaria infections across the region are attributed to the aggressive distribution of ITNs. Rather than stopping ITN distribution traditional leaders suggested various interventions including: increased education, enforcement of laws and rules through closer cooperation between the BRE and the central government agencies against misuse of ITNs and the use of small mesh nets in general, as well as increased monitoring and a program to remove older or extra nets from the population were cited. Some traditional leaders also mentioned the need for development of fish ponds to increase the supply of fish and alleviate the pressure on the wild fish catch. Others emphasized the need to diversify the protein sources in the diets of the lower income population away from fish and toward pulses (beans, lentils, chickpeas, etc) and dairy and meat products. From agency personnel some of the suggestions for reducing the negative impact on fisheries included stricter audits of the supply chain, requiring individuals to return an ITN to obtain an ITN (and look for evidence of misuse at this time), stricter enforcement of laws restricting use of ITNs and small mesh fishing nets, educational campaigns and closer cooperation with traditional leaders in all these efforts.

### Desk review of surveys

**Fig 6:**
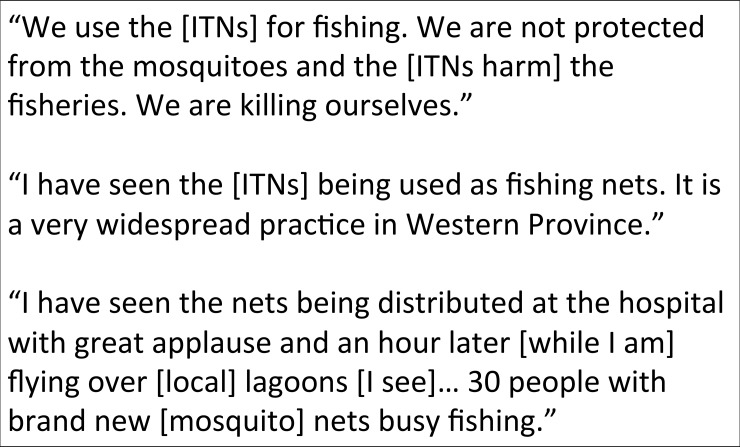
Selected quotes from interviews with traditional leaders from the BRE and agency personnel.

**Fig 7:**
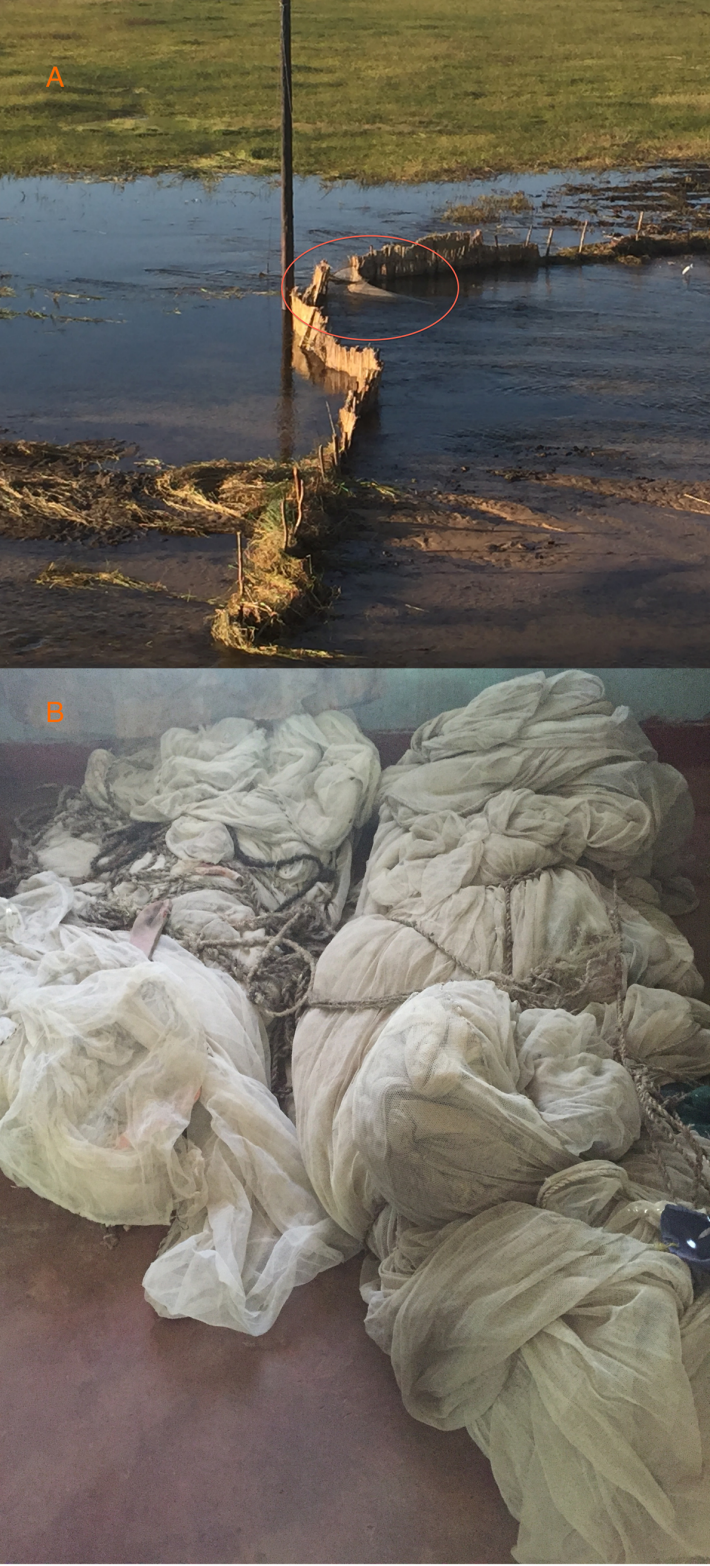
Different fishing methods incorporating mosquito netting. Single mosquito nets can be used (A—orange circle highlights ITN meant to catch fish leaving the floodplain); Many mosquito nets can be sewn together as exhibited by these confiscated nets made up of more than 50 ITNs each (B).

From the review of surveys, the consumption of fish in the area has declined by more than 50% from 2007 to 2013 (Fig 6). Improvement in child nutritional status observed early in the century has also stalled. Stunting initially declined 2000–2007, however 2007–2013 saw no change in the prevalence of children with stunted growth (Fig 7).

## Discussion

There is little doubt that ITNs, (both old and new) are used as fishing nets in the Western Province of Zambia. There are great questions about what this behavior means for human health and the strength of the fisheries. From the desk review of surveys, malaria has not decreased, decreases in malnutrition have stalled, and consumption of fish and protein decreased. We urge caution in linking these trends back to the behavior of mosquito net fishing. The diversion of ITNs meant for disease control to fishing may be correlated with a relatively recent rise in the prevalence of both anemia and malaria infections, as well as a stall in the decline of stunting of children in the region, however further investigation is required.

In the floodplain, mosquito net fishing potentially interacts with seasonality to exacerbate negative effects on fisheries in the region. Every year as the dry season progresses and the flooded river retreats to oxbow lakes and pans, fishing becomes easier as fish are concentrated in these water bodies. The concentration of fish combined with small mesh nets remove larger percentages of all fish types and sizes, a problem of overfishing from both a balanced fisheries perspective and size-selection perspective. Although these observations need to be confirmed through systematic data collection of fish size and prices in local markets, it is telling that all the traditional leaders and all the agency personnel had observed that fish catches were lower and fish prices higher at the same time that the confiscations of mosquito netting had drastically increased.

Stopping the free distribution of ITNs may slightly improve the fisheries situation, though such drastic measures must be balanced against the community-level benefit of ITN coverage [7,8]. Furthermore ITNs were only a part of the problem of unsustainable fishing–nets made of shade cloth, “chitenge” (bed sheet) cloth, or even small gauge net sizes are also widely used in the area [38]. The use of small mesh fishing nets preceded the distribution of ITNs, however widespread distribution of ITNs has facilitated rapid expansion of this destructive fishing practice.

Although this research took place only in Western Province, Zambia, the use of ITNs for fishing may be more widespread as some of the interviewed agency personnel indicated that ITNs are being misused as fishing nets in a variety of other countries in sub-Saharan Africa. The current intervention paradigm of indiscriminately distributing free ITNs en masse every few years must change. Purposeful ITN distribution including such schemes as “return a net to get a net” or continuous public health surveillance to ensure nets are being used correctly in the households is needed to ensure the health of the fisheries and ultimately the health of the population.

Some of the subjects interviewed indicated a link between in-migration and fish poaching with use of small mesh nets, including ITNs, and meeting the demand for fish originating from non-local markets. The term poaching is not often used in conjunction with fishing, but is an apt description of the problem. Exporting poached fish across borders or to urban centers such as the Copperbelt and Lusaka necessitates a role for fish traders or other parties linked to larger-scale distribution networks.

At its core the misuse of ITNs for fishing nets appears to be a problem of livelihoods and local governance. The importance of fish as the ‘bank in the water’ for poor rural residents cannot be overstated [25]. International donors have an important part to play in ensuring that countries receiving ITNs do not see subsequent crashes in fishery strength as a result of widespread mosquito net fishing. Incorporating funding for fishery management or education programs would likely help avoid unintended consequences of aid. At the same time local governance, i.e. traditional leadership, plays a large role in the subsistence farmers’ use of the land in sub-Saharan Africa where a vast majority of land is communally owned and administrated by chiefs and traditional leaders [33,39,40]. Empowering traditional leaders with the appropriate authority to enforce fishing regulations would likely lead to healthier fisheries. Including traditional leaders in the distribution of ITNs would also benefit, and could lead to improved ITN coverage as has been witnessed in other interventions in Zambia [41]. Fisheries management suggested setting aside areas where fishing is not legal at any time of the year in order to provide shelters where fish populations could breed and flourish to restock the fisheries, an intervention that has had success in many other contexts [42]. Such protected areas would be in addition to the current policy of implementing the national “fish ban” where the banning of all fishing takes place in Zambia from December 1 to the end of February to try and allow fish stock regeneration during the peak breeding season.

The in-depth interviews with key informants are a particular strength in this study. In this case, we believe that recording the observations, experiences and attitudes of the individuals with the responsibility of regulating fisheries in the region is valuable for understanding the extent to which ITNs are utilized as fishing nets. Furthermore, illegal behavior, such as mosquito net fishing, is often hidden and can be challenging to document. Asking key informants if a practice is widespread in a community is one way to obtain information on illegal behavior. The traditional leaders interviewed herein are also farmers who regularly fish. The interviewing of key informants is unfortunately unable to quantify how often ITNs are used as fishing nets as was done on the shores of Lake Tanganyika where mosquito net fishing was also found to be widespread [43].

## Conclusions

Mosquito net fishing appears to be widely practiced in Western Province, Zambia. The donor community should take seriously the misuse of ITNs for fishing nets and employ a proactive approach to addressing the underlying issues and causes. Malaria control or even elimination will be challenged by the problem of misuse of ITNs and programs should address the issue. Ignoring the problem may lead to ecological degradation and increased food insecurity [31,44].

## Supporting information

S1 File Excel spreadsheet of survey data retrieved from publicly available nationally representative 2-stage cluster surveys. (XLSX)

S2 File Field notes from qualitative interviews. (DOCX)

## Abbreviations

ITN: insecticide-treated mosquito net
