# Supplementary material for: Widespread mosquito net fishing in the Barotse floodplain: Evidence from qualitative interviews
Source: PLoS One. 2018 May 2;13(5):e0195808. doi: 10.1371/journal.pone.0195808 (PMC5931466; doi:10.1371/journal.pone.0195808)
Supplement: S2 File — (DOCX) [file pone.0195808.s002.docx]

**Notes from Interviews**

Traditional Leaders

All the traditional leaders believed that fish is a critically important source of protein for the people of the Western Province. In addition, they were familiar with the target species for fishermen in the area. Excerpts from the interviews illustrate the point:

*“Here you cannot talk about eating nshima [local corn-based dietary staple] without fish. It is an important component of our diet.”*

*“From time immemorial, in this [region] the Losis [the local ethnic group] are known…to love fish.”*

*“[For] the Losis,…their life, their upbringing…is fish. That is daily consumption for the Losis.”*

*“People rely on fish for food and income [in this area].”*

The traditional methods for catching fish were also well-known by the leaders and included small and large baskets, trident type spears, fish traps and nets with mesh of varying size depending on the size of the target species- e.g., 3.5”, 3” and 2.5”.

Traditional methods are designed to be selective to allow the escape of very small juvenile fish to keep the fish stock replenished. Leaders went to great pains to emphasize that such methods have been in use for at least hundreds of years and had not resulted in problems with declining fish populations from overfishing.

When asked about the current and expected future health of the fisheries, leaders were pessimistic. Fish populations were believed to be declining and fish prices increasing for all types. Primarily the leaders observed the decline in the size of the fish caught as well as the fewer numbers. For instance:

*“The fish stocks are completely depleted. The size is terrible. We measure it with our hands and now it goes down to the fingers [in size]. They are so small you now dry the fish whole, without opening them. The situation needs urgent and drastic measures.”*

*“The fishermen are saying there are no fish left in the plains.”*

*“As for now, our fishing is going down.”*

*“In two years, there will be no fish.”*

In general, the leaders attributed this to three main factors: drought, population increase and use of very small mesh nets for fishing, including but not limited to ITNs. In fact, leaders described the use of various unorthodox materials as fishing nets in addition to ITNS such as shade cloth and even bed sheets.

The factors were also interactive. In a prolonged drought situation, fishing becomes easier as fish are concentrated in the remaining water bodies. Concentration combined with small mesh nets remove larger percentages of all fish types and sizes. Contrariwise, sustained rains, especially over several seasons, makes fishing more difficult as the fish can disperse across larger areas and breed without being disturbed.  Fish stocks can replenish themselves.

Also, leaders explained that the use of small mesh fishing nets preceded the distribution of ITNs. And some leaders indicated that it was primarily new arrivals to the area, rather than the long-term resident fishing populations which introduced the small mesh fishing nets- known locally as sefa sefa or komba komba. However, as ITNs became available, they were also used as sefa sefa or incorporated into sefa sefa.

The leaders explained that sefa sefa, including ITNs, can devastate fisheries. This happens because, unlike traditional methods, the holes are not large enough for smaller fish and juveniles of larger species to escape capture. When such small fish are removed from the fishery, the sustainability of the fishery is imperiled because the food supply for large fish is depleted and the juvenile fish do not grow to adulthood. Over time, fishing in this manner will deplete a fishery. This negative dynamic was well understood by the leaders and articulated consistently by them.

When asked specifically about the use of ITNs, leaders indicated that new nets and older nets were used, either individually, or sewn into larger nets. The larger nets could be composed solely of ITNs or include ITNs along with small mesh fishing nets and other materials. Leaders also observed that at times newly distributed ITNs were diverted from mosquito deterrence and used only for fishing. Other times a household might have extra ITNs such that household members could be protected from mosquitoes and the ITNs could be used for fishing.  Since ITNs are distributed for free and in very large numbers, they are readily available for many uses.

*“We use the [ITNs] for fishing. We are not protected from the mosquitoes and the [ITNs harm] the fisheries. We are killing ourselves.”*

*“I have seen the [ITNs] being used as fishing nets. It is a very widespread practice in Western Province. I would be very surprised if you ask any of these local people if he or she would say they have not seen them [ITNs used as fishing nets].”*

*“They are using the mosquito nets in their houses, and when they receive a new net, they use the other one for fishing.”*

Despite the number of problems raised with the use of ITNs, leaders were consistently opposed to stopping their free distribution. The dramatic reductions in malaria infections across the region were attributed to the aggressive distribution of ITNs. Instead of ending distribution leaders emphasized increased education, enforcement of laws and rules through closer cooperation between the BRE and the central government agencies. Increased monitoring and a program to remove older or extra nets from the population were also cited.

*“The number of mosquitoes is going down and malaria is going down [due to the distribution of ITNs].”*

*“You should continue to disburse them. You have to disburse them. But the distribution should not only be through health centers. There are people who cannot access the nets from health centers…… Can you add the chiefs as part of the distribution networks? I can ask my ndunas [chiefs] to bring me a list of people to whom they have given [ITNs]. We can then do an audit.”*

*“We are working hand-in-hand with African Parks to not only protect the wildlife but also the fish.”*

*“Let the government rush to us when we catch people using the nets- not what we are doing now. We have been holding meetings, but it seems we are not achieving anything.”*

Some traditional leaders also mentioned the need for development of fish ponds to increase the supply of fish and alleviate the pressure on the wild fish catch and provide another source of income generation for the region (see Musumali, Heck and Simon, 2009). Others emphasized the need to diversify the protein sources in the diets of the lower income population away from fish and toward pulses and dairy and meat products. This would require expansion of production in these sectors and a shift in the cultural foodways of the local populace through education and outreach efforts.

Agency Personnel

Interviews with Ministry and African Parks personnel revealed that all were aware of the campaign to distribute ITNs on a very large scale in Zambia and Western Province. In addition, all had either witnessed or heard about the misuse of ITNs as fishing nets. They agreed with the traditional leaders that ITNs could be used individually, as part of fish traps in place of traditional baskets with larger holes, or as part of larger nets which might contain a number of materials. They also agreed that long-term residents engaged in fishing were less likely to use small mesh nets of any composition than immigrants to the area.

During our visit we were shown two very large nets composed of 50 ITNs each sewn together. The nets were confiscated from fishermen in the area. Since ITNs are transported for distribution in 50 net bundles, agency personnel believed that the supply chain of ITNs might be corrupted. That is, large numbers of nets might be diverted before their distribution to families or individuals and end up as fishing nets (see Image 1). At least on traditional leader also indicated that the ITN supply chain might be corrupted at times.

*“It is almost two weeks now, our [Fisheries] officers in cooperation with African Parks,…they went into the field and they confiscated illegal fishing gear and these are the mosquito nets which have been joined together…probably more than 50 meters.”*

*“The Health Ministry is not distributing to individual in bundles. Maybe you give four or so [individual nets] to a household depending on how big that family is. So for those particular individuals [the Ministry apprehended] I think they are pilfering [the nets] from some storage points.”*

*“Both the old ones and the new ones are being used... In most cases they use the new mosquito nets.”*

*“Old nets don’t work for fishing- they break.”*

*“I can show you photos of nets over a 100 meters long and 6 meters wide with the…Ministry of Health logo attached to each net. [They use these nets] as drag nets. If we confiscate nets, individuals will use other materials such as shade cloth.”*

*“I have seen the nets being distributed at the hospital with great applause and an hour later [while I am] flying over [local] lagoons [I see]…30 people with brand new [mosquito] nets busy fishing. I have photo proof of this.”*

And all agency personnel agreed that the misuse of ITNs as fishing nets were having a negative impact on fisheries in the area. Fish catches were believed to be declining and the size of fish for sale in the local markets was diminishing in size and number and increasing in price. However, these observations need to be confirmed through systematic data collection of fish size and prices in local markets. Still, it is telling that all the traditional leaders and all the agency personnel had observed that fish catches were lower and fish prices higher. One agency employee indicated she did not purchase fish for personal consumption any longer because “it is too expensive for me.”

*“We have not done studies on numbers of fish in a pond, pan or lagoon post [mosquito net distribution]. What I see on the ground is the size of fish late in dry the season is hugely impacted. Where we have confiscated a lot of nets, people complain about no fish late in the season.”*

*“The netting is linked to the increase in trade [outside the region]….All this fish is going to the copper mines, to Congo…to the city centers. There is a market for fish early [in the season]. You have high unemployment and young guys looking for a way to make money.”*

*“When these [ITNs] go into the river, they are collecting basically everything which is under the water- even the small fish which is not supposed to be taken out of the river. In the long run they are having an impact on fishing production.”*

Like the traditional leaders, the agency personnel agreed unanimously that the distribution of ITNs on a large-scale should continue. They recognized the importance of the ITNs in reduction in malarial infections. However, they also saw the need for alterations in the distribution process. Some of the suggestions for reducing the negative impact on fisheries included stricter audits of the supply chain, requiring individuals to return an ITN to obtain an ITN (and look for evidence of misuse at this time), stricter enforcement of laws restricting use of ITNs and small mesh fishing nets, educational campaigns and closer cooperation with traditional leaders, fish traders and the media in all these efforts. One fisheries staff person believe the media should cover the issue “twice per week.”

*“It [ITN distribution] should be continued. Malaria and the irritating nature of the mosquito is…you can’t sleep at night. Malaria is the number one cause of death in children under the age of five. We need [the nets] but you need careful sensitization programs and follow-up operations otherwise you are being irresponsible.”*

*“The distribution of the nets…should continue. [But] most of the people are not aware that the nets are meant to protect them from mosquitoes. Malaria kills more than any other disease in Africa.”*

*“Other stakeholders along with the Ministry of Health and the Department of Fisheries should find a way to work together to sensitize the community. The Ministry of Health has not taken an interest or go to see whether their nets are really used for the intended purpose.”*

*“If you could say no one can trade [sell in the market] [the] very small fish it would have a chain effect so that those persons using the [ITNs and other small mesh nets] would not use them. But, it is a touchy issue. It has been tried before and there was no political will.”*

*“Some people do not believe mosquitoes cause malaria. To them malaria is caused by a curse. So they will not let the children sleep under the nets.”*

*“The [traditional] leaders are in charge of certain water bodies. In order for fishery personnel to have access to them it can be difficult. We have tried village management conservation committees. Last year we formed about five. The committees face a lot of challenges. In order to continue working they need support. They receive no help from the Department of Fisheries. They do not have authorities to apprehend [those violating the law] and process the case. The Department of Fisheries and the Barotse Royal Establishment need to iron out these issues… If these are done, then I see a successful [ending].*

The agency personnel were less likely to suggest the development of fish ponds to increase fish supply and reduce the pressure on wild fish populations. However, they did suggest setting aside areas where fishing is not legal at any time of the year in order to provide shelters where fish populations could breed and flourish to restock the fisheries. Such protected areas would be in addition to the current policy of banning all fishing in the Zambezi River and adjoining water bodies from December 1 to the end of February.

**Figure 1: Rank of Traditional Leaders in Western Province, Zambia**

KING

SENIOR CHIEFS/QUEENS

CHIEFS

SUB-CHIEFS

VILLAGE HEADMEN

**Source: Lwambi Royal Establishment, 2015; Interview Results**
